# Different Approaches to Caponisation of Cockerels and Their Relation to Welfare

**DOI:** 10.3390/ani16030355

**Published:** 2026-01-23

**Authors:** Alenka Dovč, Jurij Žel, Gordana Gregurić Gračner, Marko Cvetko, Veronika Budin, Zoran Žlabravec, Primož Klinc

**Affiliations:** 1 Institute of Poultry, Birds, Small Animals and Reptiles, Veterinary Faculty, University of Ljubljana, Gerbičeva 60, 1000 Ljubljana, Slovenia; zoran.zlabravec@vf.uni-lj.si; 2 National Centre for Animal Welfare, Veterinary Faculty, University of Ljubljana, Gerbičeva 60, 1000 Ljubljana, Slovenia; 3 Small Animal Clinic, Veterinary Faculty, University of Ljubljana, Gerbičeva 60, 1000 Ljubljana, Slovenia; jurij.zel@gmail.com; 4 Department of Animal Hygiene, Behaviour and Animal Welfare, Faculty of Veterinary Medicine, University of Zagreb, Heinzelova 55, 10000 Zagreb, Croatia; ggracner@vef.unizg.hr; 5 Institute of Pathology, Wild Animals, Fish and Bees, Veterinary Faculty, University of Ljubljana, Gerbičeva 60, 1000 Ljubljana, Slovenia; marko.cvetko@vf.uni-lj.si; 6 Veterinary Hospital, Vipava, Gradiška Cesta 10, 5271 Vipava, Slovenia; veronikaabudin@gmail.com; 7 Experimental Centre for Domestic and Laboratory Animals, Clinic for Reproduction, Veterinary Faculty, University of Ljubljana, Gerbičeva 60, 1000 Ljubljana, Slovenia

**Keywords:** chickens–surgery, cockerels, caponisation, animal welfare, corticosterone

## Abstract

The aim of this study was to improve surgical caponisation and identify stress factors associated with different pain management methods and post-operative periods. A total of 72 Prelux-G cockerels were caponised at six weeks of age. Eight experimental groups were established based on caponisation status and pain management. Four groups were caponised using different pain management protocols (none, anaesthesia only, analgesia only, or both), while four non-caponised groups served as controls, including one group that received anaesthesia and analgesia without surgery. The remaining three groups served as non-caponised controls with varying levels of handling. The researchers monitored body weight, feed intake, respiratory and heart rate, vocalisations, response to pain, and depth of anaesthesia. The results confirmed that caponisation is painful, as reflected in increased physiological responses and vocalisations. The use of non-steroidal anti-inflammatory drugs alone did not provide effective pain relief. Additionally, the surgical procedure had no significant effect on weight gain or feed intake during the seven days following surgery. Stress levels, measured by blood serum corticosterone concentration, also increased during the procedure. The study concludes that anaesthesia significantly reduces pain and facilitates surgery, which is essential for improving animal welfare.

## Simple Summary

Caponisation is a surgical procedure performed in the coelomic cavity of cockerels to produce high-quality meat valued for its distinctive aroma and tenderness. In many countries, the procedure is still carried out without anaesthesia or analgesia, raising serious animal welfare concerns. These concerns have led to its prohibition in several countries. Where caponisation is permitted, it must be performed professionally and in strict accordance with animal welfare standards. Numerous studies have shown that roosters experience significant pain, reduced appetite, and behavioural changes following the surgical procedure, indicating that the procedure has substantial welfare implications. Immunocastration is considered a more humane alternative; however, its widespread use depends largely on consumer acceptance. Male chicks from layer breeds are considered a by-product of the poultry industry not only because they cannot lay eggs, but also because rearing them for meat is often considered economically unviable. For this reason, males are often culled after hatching. However, this procedure also presents an opportunity to use male chicks from layer breeds. The aim of our study is to determine whether the use of anaesthesia and analgesia can improve animal welfare and provide an ethical justification for the continued practice of surgical caponisation with adequate pain management.

## 1. Introduction

Caponisation is a surgical procedure performed in the coelom of cockerels to obtain high-quality meat, which is considered a speciality due to its aroma and succulence. The meat is rich in nutrients and is believed to be more tender, juicier, and more flavourful than that of intact cockerels [1]. The procedure involves the removal of the testes in male chickens and can also be achieved chemically through immunocastration [2].

Immunocastration can be used as an alternative. Gonadotropin-releasing hormone-(GnRH) immunocastration minimises the stress experienced by cockerels, reduces the risk of infection and complications associated with surgery, and significantly improves animal welfare. In addition, there is no risk of drug residues, making it suitable for use in production. Several commercial immunocastration products are used on some farm, domestic, and wild animals (wild boar, deer) [3]. However, there is no commercial product available for poultry. Quaresma et al. found that immunocastration of cockerels with Improvac could be an alternative to caponisation with significant improvements in animal welfare [2]. Wang et al. and Zeng et al. reported that cockerels vaccinated with Improvac showed measurable effects on muscle development; however, these effects were limited, as immunocastration influenced thigh muscle development but had little or no effect on pectoral muscle growth [4,5].

Within the European Union, immunocastration is regulated under veterinary medicinal product legislation and is classified as a veterinary medicinal intervention. Its use requires authorised immunological veterinary medicinal products and must be carried out in accordance with applicable regulatory requirements, including veterinary supervision. At present, no immunocastration products are authorised for routine use in poultry within the EU [6].

Although most commercially raised chickens today are bred for rapid growth and high feed conversion, capons represent a niche segment typically aimed at gourmet markets and certain cultural traditions, particularly in southern Europe and parts of Asia [1]. The procedure is not limited to broiler chickens; it has also been performed on male chickens of layer breeds [1,7,8], pheasants [9,10], turkeys [11], other domestic poultry [12,13], and even pigeons [14]. Studies have shown that capons generally exhibit calmer behaviour, better feed conversion, and higher fat storage, resulting in better meat quality [7].

Historically, caponisation dates back to Roman times and was widely practised throughout Europe and Asia. In modern poultry production, this practice offers a potential use for male chickens of laying breeds, which are otherwise considered an economic by-product and are often culled shortly after hatching. Surgical castration allows these males to be raised for meat, reducing biological waste and improving resource efficiency [1].

While some authors deny that capon castration causes significant pain and argue that the use of anaesthesia and analgesia during the procedure is unnecessary or ineffective in reducing distress [15], others report that surgical castration of capons may cause severe pain and stress responses and therefore propose alternative approaches, such as immunocastration or production of intact males [2,3]. In addition, certain animal welfare and animal rights organisations argue that elective surgical procedures performed solely to enhance meat quality raise ethical concerns and should be avoided [15].

Experienced caponisers work very rapidly, castrating approximately 200 birds in one hour [16]. Due to the location of the testes alongside the dorsal aorta and the posterior vena cava, accidental damage to either of these major blood vessels during caponisation may result in rapid and often fatal intra-coelomic haemorrhage [16,17]. Reports of orchidectomy in roosters indicates that mortality can be very high, primarily due to intra- and postoperative haemorrhage (ranging from 25% to 41.5%, depending on the surgical approach) [16]. If antiseptics or antibiotics are not used-and even in some cases where they are-infection may develop. If aseptic technique is not used during the procedure, the risk of infection increases significantly. Notably, postoperative infections can occur despite strict adherence to aseptic surgical protocols, both in animals and in humans, with clinically relevant incidence rates [18]. If infected wounds do not heal properly, cockerels may suffer and eventually die. A condition caused by air accumulation in the subcutaneous tissue as the wound heals occurs sporadically [16].

Prolonged pain and stress may result in immunosuppression, impaired growth performance, and behavioural alterations. In poultry, pain perception involves neurological and biochemical mechanisms comparable to those described in other avian species and mammals. Poultry possess nociceptors—specialised sensory receptors that respond to potentially harmful stimuli such as mechanical injury, extreme temperatures, and chemical exposure, enabling the perception of pain and stress [19]. Ethical considerations related to surgical procedures have therefore become an important aspect of animal welfare discussions. Acute pain responses following surgical interventions have been described, including vocalisations, elevated physiological parameters (heart rate, respiratory rate, and body temperature), reduced feed intake, and changes in social and exploratory behaviour, all of which are recognised indicators of suffering [20].

Public opinion is increasingly critical of invasive procedures in animals, especially when less harmful alternatives exist. For example, consumer surveys in several European countries indicate a growing rejection of surgical procedures on farm animals unless they are medically necessary or demonstrably beneficial to the animal’s welfare [21].

Immunocastration, which uses a vaccine to block GnRH, has emerged as a potential welfare-friendly alternative. Although it is currently more common in pig production, recent trials have also investigated its use in poultry. Preliminary results suggest that immunocastration can reduce aggression and improve meat quality without the trauma associated with surgery. However, consumer acceptance remains limited, partly due to concerns about hormones or residues, despite scientific assurances to the contrary [3].

According to Council Directive 98/58/EC concerning the protection of animals kept for farming purposes, animals must be protected from unnecessary suffering and surgical procedures must be carried out under appropriate conditions, including the use of anaesthetics and analgesics [22]. In addition, only trained and certified persons may perform such procedures. Descriptions of surgical castration of roosters often differ [14,23,24,25,26,27,28]. Furthermore, research frequently addresses pain in birds during surgical castration or other painful conditions [14,25,26,27,28,29].

Despite these regulations, caponisation remains controversial. In several European countries, including Austria, Germany, the Netherlands, and Sweden, the practice is banned outright [30,31,32,33]. In France, Italy, and Spain, where capon production is of cultural and economic importance, it is still permitted under strict regulation [34,35,36]. In Belgium, Croatia, Poland, and Slovenia, surgical caponisation is only permitted if performed under anaesthesia [37,38,39,40].

In the United Kingdom, caponisation has been banned since 1982, although the import and sale of capons remain legal [41]. In the United States, there is no federal legislation on the subject, and regulations vary by state. In some states, the practice is explicitly prohibited, while in others, there are no specific regulations regarding caponisation [16].

Commission Regulation (EC) No. 543/2008 also stipulates that capons must be surgically castrated before reaching sexual maturity and slaughtered at minimum age of 140 days. After surgery, capons must be fattened for at least 77 days to ensure suitable meat characteristics [42].

A hundred years ago, the birds were kept without food and water for 36 h before the surgical procedure. They were to be caponised at 7–11 weeks of age for light breeds or 10–14 weeks for heavy breeds [43].

Cockerels may be caponised at different ages, most commonly between 6 and 14 weeks, depending on breed and body weight. An optimal weight of around 0.5 kg is generally recommended. Fast-growing breeds (e.g., broilers) are usually castrated earlier, while slow-growing traditional breeds are operated on later [1].

Age at castration plays a key role in animal welfare. Younger birds generally recover faster, experience less stress during the procedure, and have better growth performance and meat quality. Studies also show that survival rates are significantly higher when caponisation is performed earlier [20].

Capons are particularly valued for their high intramuscular fat content, tender texture, and mild flavour. These characteristics are further enhanced by the absence of male sex hormones after caponisation, which otherwise influence muscle development and behaviour. Sensory studies have confirmed that capon meat has better organoleptic properties compared to intact males and females [44,45]. Malovrh et al. compared three capon genotypes–Barred Prelux, Sulmtaler and Styrian chicken–slaughtered at 163 and 198 days of age and found statistically significant differences in meat juiciness and flavour depending on genotype and slaughter age [45].

Various sources of stress are recognised, including physiological stress, psychological stress, stress arising from pathological conditions, environmental stress, and acute versus chronic stress. The situation is complex because the hypothalamic–pituitary–adrenal (HPA) axis response to stress has evolved to help animals cope with stressors in the natural world, but animals kept by humans are subjected to stressors they would not normally encounter, or are exposed to stressors such as caponisation or restrictive housing conditions for much longer periods than would naturally occur [46].

The main adrenocortical hormone and primary glucocorticoid responding to stress in birds, is corticosterone (CORT) rather than cortisol [46,47]. As adrenocortical output is commonly measured using blood samples, plasma CORT measurement is regarded as the “gold standard” not only for assessing stress responses, but also as the benchmark against which all other sample types are evaluated. Multiple plasma samples can be used to assess the reaction time, frequency, and amplitude of the HPA axis response to stress. In this context, adrenocortical output refers to the secretion of corticosterone by the adrenal cortex following activation of the HPA axis. Repeated plasma sampling can be used to assess the timing, frequency, and magnitude of HPA axis responses to stressors. However, blood sampling itself may represent a substantial stressor, thereby confounding the assessment of adrenocortical activity [46].

In recent years interest in faecal glucocorticoid assays as a non-invasive method for monitoring stress in mammals and birds has increased substantially [48]. The limitations associated with collecting blood samples are the primary reasons for using alternative, minimally invasive sample media such as faeces, feathers, and eggs [46].

Caponisation embodies a long-standing conflict between tradition and animal welfare. Although it provides an opportunity to utilise surplus male chicks and produce meat of exceptional sensory quality, it raises serious ethical concerns due to the pain and distress associated with surgical intervention without anaesthesia or analgesia. Increasing societal awareness of animal sentience and the principles of the Five Freedoms has intensified calls for refinement, reduction, or replacement of invasive practices. Consequently, the search for the use of anaesthesia and analgesia–painkillers–and welfare-friendly alternatives, such as immunocastration, is becoming essential for ensuring ethical and sustainable poultry production [2,3,15,21].

This study aimed to evaluate the welfare impact of surgical caponisation in cockerels by comparing physiological and behavioural indicators of pain and stress under different perioperative protocols. We specifically assessed respiratory rate, heart rate, vocalisation and behavioural reactions during key procedural stimuli, alongside corticosterone dynamics as an endocrine marker of stress. Overall, the objective was to determine whether anaesthesia and/or analgesia meaningfully reduces pain-related responses and improves procedural conditions during caponisation.

## 2. Materials and Methods

The experiment involved 72 cockerels of the Prelux-G breed aged 6 weeks and weighing 600–700 grammes. The cockerels were divided into groups of 8 (four groups) and 10 (four groups).

Six days after caponisation, total feed intake was measured once daily (in the evening) in each experimental group. In addition, weight gain was monitored on the 25th, 32nd, 42nd (the day before caponisation), and 49th day of life (7 days after caponisation). The cockerels were fed ad libitum with BRO-FINIŠER–pellets (Emona krmila, Ljutomer, Slovenia) and water. They received water and feed via drip and hanging feeders.

All cockerels were randomly divided into 8 groups for the experiment using the GraphPad programme (GraphPad Software, San Diego, CA, USA), version from www.graphpad.com/quickcalcs/. They were fasted for 24 h, except for group 8. Fasting allows easier access for tests and simplifies the surgical procedure because the gastrointestinal tract is emptied.

In six groups (groups 1 to 6), the cockerels were fixed on the operating table to prepare them for caponisation. In two other groups (groups 7 and 8), fixation on the table was omitted to determine the effects of fixation and preoperative preparation on stress (Table 1).

In all groups (including group 8), blood samples were taken from the superficial ulnar vein to test for the presence of CORT.

### 2.1. Caponisation–Surgical Procedure

In the first six groups, the birds’ legs and wings were tightened and securely fixed to a work surface, but only the first four groups underwent caponisation. Feathers that might obscure the procedure were removed. A two-centimetre incision was made between the last two ribs on both sides. The ribs were then spread apart with a retractor, and each testicle was pulled out with a twisting motion until it detached. The incisions were not sutured. All cockerels were operated on the left side first. Group 1 received no anaesthetics or analgesics; in group 2, cockerels received only anaesthetics; in group 3, only analgesics; and in group 4, both anaesthetics and analgesics. All approaches are shown in Table 1.

The feathers were plucked from the area of the rear ribs and the skin was disinfected. A no. 11 scalpel was used to make a skin incision about 2 cm long between the posterior ribs. Immediately before the incision, the skin was pulled caudally so that it would cover the resulting opening in the coelomic cavity after completion of the procedure. The opening was made with scissors and modified Weitlaner retractor. The underlying tissue, especially the abdominal air sacs, was bluntly dissected with forceps to visualise the testicle and make it accessible. The seminiferous plexus of the testis was then fixed with Babcock forceps and the testis was removed by rotating the forceps to achieve haemostasis. The seminal vesicle usually tore off by itself; if not, any remaining attachments were sharply dissected. The wound was not sutured after removal of the testicle. The procedure for removing the testicle was then repeated on the other (right) side.

Throughout caponisation, the basic physiological parameters–respiratory rate, heart rate, vocalisations, and vocalisation in connection with the cockerels response to painful stimuli–were monitored.

The respiratory rate was determined post hoc by visual inspection of high-definition video recordings. A camera with optical zoom was mounted on a tripod and focused on the animal’s thoracic region to capture subtle respiratory movements. To ensure high measurement accuracy and minimise motion artefacts, the surgical procedure was briefly paused for several seconds at each predetermined measuring point. This allowed breaths to counted without manual manipulation of the animal, ensuring that the recordings reflected the physiological state as accurately as possible.

Heart rate was monitored by electrocardiogram (ECG). Measurements were taken during a 10-s pause after each pain stimulus, and heart rate was then determined by reading the ECG printout.

During surgical procedure, vocalisations were assessed using a simplified scoring system based on intensity (quiet, moderate, very strong) and duration (short or continuous), reflecting the immediate behavioural response to handling and procedural stimuli. Vocalisation intensity was defined based on perceived loudness and urgency, while duration referred to whether vocalisations occurred briefly or persisted throughout the observation period. The type of vocalisations was not categorised, as the behavioural observation protocol was not designed to distinguish between specific vocalisation classes. Vocalisations were assessed at control points as evaluating general response: no vocalisation (0), short, quiet vocalisation (1), continuous, moderate vocalisation (2), and very strong, intense vocalisation persisting long after the stimulus (3).

The median vocalisation scores and responses to painful stimuli were recorded in animals from groups 1–4 during the caponisation procedure. Reaction grades were defined as follows: no response (0), mild kicking or wings flapping (1), moderate kicking or wing flapping (2), strong resistance and escape attempts (3). To enhance clarity, the behavioural reaction grades were defined using an operational scoring system based on movement intensity and the need for additional restraint (Table 2).

In performing the procedure and evaluating the general response of the cockerels during surgery, we focused mainly on the following painful stimuli, which served as control points: feather plucking (F), skin incision (I), opening of the coelomic wall (O), and testicular rotation (T).

Ten minutes after the administration of anaesthetics/analgesics (groups 1–5) or after resting in the boxes (group 6), the surgical procedure was performed, lasting two minutes.

To ensure consistency and sensitivity in the assessment of physiological pain responses, statistical analysis of breathing, heart rate and reactions with vocalisation focused on a single procedural moment presumed to elicit the highest nociceptive activation–namely, removal of the second testis by rotation (T2).

Seven days after caponisation, by which time the acute stress response was expected to have subsided, the experiment was terminated by administering 2 mL/kg Exagon 400 mg/mL (VetViva Richter GmbH, Wels, Austria) into the superficial ulnar vein (vena cutanea ulnaris superficialis). One cockerel from group 1 died during the surgical procedure.

### 2.2. Corticosterone

A blood sample of 2.0 mL was taken from the superficial ulnar vein in all groups using size G21 needles. Microtubes with a separator (Becton Dickinson, Heidelberg, Germany) were used. After separation, the blood serum was pipetted into a microtube (Eppendorf tubes^®^ 5.0 mL, PP with hinged lid, LLG, Meckenheim, Germany) and stored in a freezer (at −20 °C to −24 °C). Blood serum samples were transported on dry ice to the laboratory of the Faculty of Veterinary Medicine in Vienna, Austria. CORT analysis was performed as previously described [49,50].

Blood samples (groups 1 to 4) were taken to determine CORT levels: 24 h before surgery, 15 min after completion of surgery, and 48 h after surgery. Blood samples (groups 5 to 8) were also taken at the same times as for the first four groups, according to the protocol: In group 5, blood samples were taken 15 min after the cockerels were fixed on the table, and in group 6, blood samples were taken 15 min after the cockerels had received anaesthetics and analgesics and were fixed on the table. In groups 7 and 8, the protocol schedule was followed. The order of sampling was always the same.

Blood samples were collected at three time points to capture baseline, acute, and short-term post-intervention corticosterone (CORT) responses. Sampling 24 h before the procedure provided baseline CORT levels, while sampling 15 min after the intervention corresponded to the expected peak of the acute stress response in poultry. The final sampling at 48 h post-intervention was chosen to assess recovery and the persistence of stress-related endocrine changes, as corticosterone concentrations in chickens typically peak within minutes following an acute stressor and subsequently decline during recovery [51].

### 2.3. Statistics

All statistical analyses were performed using SigmaPlot version 11.0 (Systat Software Inc., San Jose, CA, USA), which includes the integrated SigmaStat module for data processing. Before to inferential testing, data distribution was assessed using the Shapiro–Wilk test. When data followed a normal distribution (Shapiro–Wilk probability > 0.05), parametric tests were applied. In cases of non-normal distribution (Shapiro–Wilk probability < 0.05), non-parametric methods were used to ensure analytical robustness.

For comparisons between multiple independent groups, one-way analysis of variance (ANOVA) was used when assumptions of normality and homogeneity of variances were met. When these conditions were not satisfied, the Kruskal–Wallis one-way analysis of variance on ranks was applied. Post hoc multiple comparisons were conducted using either the Bonferroni *t*-test or Tukey’s test.

To compare two time points within the same group, the paired *t*-test was used for normally distributed data. For non-normally distributed data, the Mann–Whitney U test was employed. This approach ensured consistency across analyses while respecting the underlying statistical assumptions.

Descriptive statistics included calculation of the mean, standard deviation, median, interquartile range. Behavioural responses, such as reaction grades and vocalisations, were analysed as ordinal data and expressed as percentages. Group comparisons for these outcomes were conducted using non-parametric rank-based tests.

Statistical significance was defined as *p* < 0.05. All analyses were interpreted accordingly.

The cockerel that died during the procedure was treated as a missing value in the statistical analysis of individual measurements. For group feed consumption, the total weight of consumed food was adjusted to account for the reduced number of animals, ensuring the data accurately reflect the consumption of the remaining group members.

## 3. Results

Cockerels were weighed 17 and 10 days before caponisation, on the day of the procedure, and again 7 days postoperatively. Feed intake was recorded once daily for 6 consecutive days after caponisation. Table 3 shows the median body mass of the cockerels on the day of surgery and 7 days later, as well as the median feed intake during the 6-day postoperative period. Interquartile ranges and between-group comparisons are also provided. These measurements were used to assess the impact of caponisation and pain management on growth performance and feeding behaviour.

No statistically significant differences (*p* > 0.05) in mean body weight between the cockerel groups were observed 17 and 10 days prior to caponisation, or 7 days after the procedure. However, on the day of caponisation, a statistically significant difference (*p* < 0.05) was found: cockerels in group 8 (non-fasted) had a higher mean body weight than those in groups 1–7 (fasted). This difference is likely attributable to the absence of preoperative fasting in group 8.

Descriptive statistics for feed intake revealed variation between groups, with the lowest mean intake in group 4 and the highest in groups 5 and 6. Group 6 differed significantly from groups 1, 2, and 4, while group 5 differed significantly from groups 2 and 4. groups 1, 2, and 4 exhibited reduced feed intakes. In contrast, groups 5 and 6, which were not caponised, maintained higher intake levels, suggesting better overall welfare.

On the day following the surgical procedure, lower feed intake was noticed in the caponised groups compared to the uncaponised groups.

Within the surgical procedure, the designation of removal of the second testis by rotation (T2) as the peak pain point was empirically supported by multiple physiological and behavioural indicators. Respiratory rate reached its highest values at T2 across all caponised groups, with a median of 48 breaths per minute. Statistical analysis revealed significant differences between anaesthetised and non-anaesthetised animals at this stage (ANOVA, *p* < 0.001), confirming the sensitivity of this parameter to nociceptive variation.

Behavioural scoring at T2 (rotation the second testis) showed that 76.3% of animals across all caponised groups exhibited moderate to strong reactions (grades 2 and 3), confirming the high nociceptive impact of this procedural stage. Only 2.6% showed no reaction, while the remaining 21.1% displayed mild responses. These findings are consistent with previous summary data and reinforce the designation of T2 as the peak pain point.

In contrast, heart rate did not consistently peak at T2 and showed considerable variability across procedural stages.

Breathing rate was observed in groups 1–4 at the resting state before the procedure, after fixation, and at the designated peak pain point during the procedure. Statistical analyses were performed using paired *t*-tests or non-parametric alternatives, depending on the distribution and variance of the data.

Table 4 presents the average breathing rate in the observed groups before and at the designated peak pain point during the procedure. Statistical differences between the groups, as well as between the two measurements within each group are also presented.

The mean respiratory rate did not differ between groups before the procedure. Our findings indicate that respiratory rate increased significantly during the designated peak pain point in groups 1 and 3, while no such change was detected in groups 2 and 4.

Table 5 presents the median heart rate values and interquartile ranges (IQR) for each group at two control points: before the procedure (R1) and at the designated point during the procedure (T2). The data are shown as medians to ensure consistency across groups, although a normal distribution was confirmed for most datasets.

At rest (R1), groups 2, 4, and 5—those receiving anaesthesia, exhibited notably lower heart rates compared to the unanaesthetised groups 1 and 3. Statistically significant differences were observed between these two clusters, confirming the suppressive effect of anaesthesia on baseline heart rate.

Despite these intergroup differences, no statistically significant change in heart rate was detected within individual groups when comparing values between R1 and T2. This indicates that, although anaesthesia influences overall heart rate levels, the physiological response to the procedure itself did not result in a measurable increase or decrease in heart rate within any single group.

Table 6 presents the median vocalisation scores and behavioural reactions observed in animals from groups 1–4 during the caponisation procedure. The table also includes the percentage of procedures classified as difficult due to excessive movement or other disturbances caused by the animal.

Groups 3 and 1 experienced the highest proportion of difficult procedures (40% and 30%, respectively), while groups 2 and 4—both anaesthetised—had fewer interprocedural complications (0% and 10%, respectively). This trend is consistent with the observed reaction grades and vocalisation scores.

Unanaesthetised groups (1 and 3) showed a higher percentage of strong reactions and designated the point in the procedure (T2), as well as strong vocalisations and strong reactions across all measured points. These points included feather plucking, skin incision, and testicular rotation on both sides.

The data suggest that anaesthesia reduces both vocalisation and physical resistance during surgical manipulation, thereby improving procedural conditions and animal welfare.

In addition to vocalisation and reactions, anaesthesia without pain stimuli was evaluated based on decreased muscle tone and absence of movement prior to the procedure. A grading scale from 0 to 3 was used: grade 3: the animal was fully awake; grade 2: the animal was dormant with some reactions; grade 1: the animal was asleep with occasional reactions; grade 0: the animal was fully anaesthetised.

All animals without anaesthesia were graded as fully awake (grade 3). In group 2, 40% of the animals were graded as 1, and 30% as 2. In group 4, 20% were graded as 1, 20% as 2, and 10% as 3. In group 5, 63% of the animals were graded as 1. The remaining percentages in all groups were graded as 0, indicating anaesthesia.

**Figure 1:**
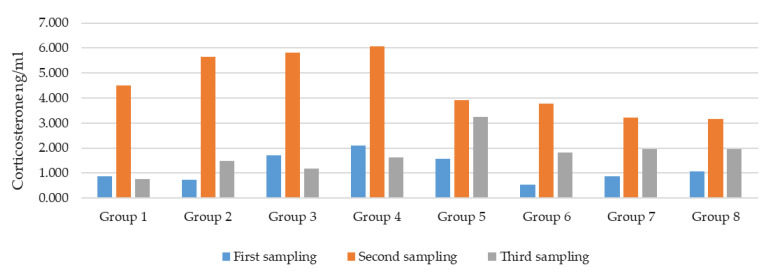
CORT concentration (ng/mL blood serum) in the different groups (N = 63).

Table 7 and Figure 1 show the median CORT concentrations (ng/mL blood serum) and interquartile ranges for all experimental groups across three sampling points. The table also indicates statistically significant differences between samplings within each group (*p* ≤ 0.05), reflecting the hormonal response to procedural stress.

CORT concentrations were measured at three sampling points to assess the physiological stress response. In all groups except group 8, the second sampling yielded significantly higher values than to the first (*p* ≤ 0.05), indicating an acute stress reaction during any procedure.

In groups 1, 2, 3, and 4 the second sampling showed the highest CORT levels, with statistically significant differences compared to both the first and third samplings. In groups 6 and 7, the second sampling was significantly higher than the first, while no difference was observed between the second and third. Group 5 showed elevated values at both the second and third samplings compared to the first. In group 8, no significant differences between samplings were detected.

These results indicate a hormonal response to procedural stress, with peak CORT concentrations occurring during the caponisation and partial recovery observed thereafter.

## 4. Discussion

Caponisation represents a niche yet culturally embedded practice within European poultry production. It is traditionally associated with specific regional products of high culinary value but raises contemporary concerns regarding animal welfare and ethical acceptability. The regulatory landscape across Europe varies considerably: Some Member States have imposed outright bans on non-therapeutic surgical procedures, others allow the practice under strictly controlled conditions (typically requiring anaesthesia and veterinary oversight), while in some countries it persists within traditional or protected product frameworks.

When properly performed, under anaesthesia and analgesia by qualified personnel, surgical caponisation can be ethically justified and may potentially improve animal welfare. This is especially relevant for male chickens of laying breeds [7].

In current livestock management in some EU countries, castration procedures—particularly in species such as poultry—are often not classified as surgical interventions. As a result, they are frequently carried out by personnel trained primarily in the mechanical execution of the procedure, without formal education in veterinary surgery or a comprehensive understanding of anatomical structures and physiological processes.

Unfortunately, legislation in some countries is not strictly enforced, and caponisation is still often performed without the use of anaesthesia and analgesia. This practice raises significant concerns regarding the lack of aseptic technique, inadequate pain management, and compromised animal welfare standards. The normalisation of such procedures without proper oversight highlights the urgent need to reclassify them as invasive interventions and to establish clear, evidence-based guidelines that ensure ethical and professional standards are upheld. For these reasons, we have investigated the impact of anaesthesia and analgesia during caponisation to evaluate the benefits of incorporating pain management protocols, compared with the traditional approach performed without anaesthesia.

It is estimated that the death rate during caponisation is about 3 to 5%, depending on whether the procedure is performed by an experienced or inexperienced person. In older cockerels, mortality reaches up to 50%, and such an intervention is also more time-consuming and professionally demanding [1,16]. The observed mortality during the procedure or in the postoperative period was 1.4% (one cockerel from group 1; the surgical procedure was performed without anaesthesia and analgesia). The cockerel died during surgical procedure. There was extensive bleeding in the coelomic cavity. The lower mortality of caponised cockerels during and after the procedure could be attributed to better working conditions, including experience of the surgeon, improved hygiene, sterility of the procedure, slower course of the procedure and the use of anaesthesia. We also believe that this situation can be achieved in practice with minimal financial investment.

During fasting, cockerels should be kept in cages or on bare floors; otherwise, they will satisfy their hunger by eating litter [52]. Long fasting periods and drastic environment changes raise concerns about animal welfare, which is also a reason the procedure is banned in many countries.

Protocol of this study followed the general consensus is that cockerels should be adequately prepared by withdrawing feed for 24 h and water for several hours before the surgical procedure, so the intestines are not full and crowding in the abdominal cavity is minimised. Empty intestines facilitate access to the reproductive organs and reduce the frequency of bleeding during the removal of the testicles, and thus the death of the capon after the procedure [1]. In our study, we followed the protocols described above [1,52].

The literature describes a short recovery period after surgical procedures and lower body weight immediately afterwards most likely associated with fasting before caponisation and stress during the procedure. It has been found that, although feed intake is reduced immediately after the procedure, both feed intake and weight gain return to the same or even higher levels overall compared to uncastrated cockerels [53,54].

A significant transient decrease in body mass due to fasting was also confirmed in our study. Caponisation did not affect body mass gain in the first six days after caponisation. A trend towards lower feed intake was observed after the procedure in the caponised groups compared to the non-caponised groups, including those animals that underwent anaesthesia, although there was no consistent pattern of significance across groups. Although this suggests a potential impact of caponisation on early postoperative feed consumption, daily statistical comparison was not possible because only a single measurement per group was available.

Feed intake from day 1 to day 6 was reduced in three out of four caponised groups (groups 1, 2, and 4), suggesting that the procedure itself may impact postoperative consumption regardless of anaesthesia. Based on comparative analysis, the influence of anaesthesia on reduced feed consumption can be largely ruled out.

The absence of a statistically significant difference in some groups could imply a potential benefit of non-steroidal anti-inflammatory drug (NSAID) administration; however, this interpretation is not supported by results from group 4, which also exhibited significantly lower feed intake. It is important to note that the cockerels received only a single dose of a non-steroidal analgesic, which was likely insufficient for effective postoperative pain management. A regimen involving prolonged administration of analgesics via drinking water would likely represent a more appropriate strategy and could better elucidate the role of NSAIDs in improving welfare outcomes following caponisation. Feed intake is also a sensitive welfare indicator, and its suppression in stressed animals underscores the broader impact of procedural distress beyond immediate physiological responses.

An additional factor that may have influenced feed intake measurements is the inability to account for feed scattering by the cockerels, which could have introduced variability into the recorded consumption data. This limitation should be considered when interpreting the results, as it may have largely masked differences between groups.

There are several reasons for the conflicting results on growth and feed intake between different studies, including differences in the age of animals at caponisation and slaughter, breeds or genetic lines, and diets and breeding conditions [55,56].

Unlike the above-mentioned studies, we monitored body weight and feed intake only for one week after the procedure, and we did not monitor feed intake individually, nor did we account for possible dispersion. An additional variable is the breed type used and the breeding conditions, which differ from those in the previously mentioned studies.

Because we did not find any guidelines in the literature on the anaesthetics or analgesics used in caponisation, we prepared our protocol with the most commonly used drugs and their doses. The pharmaceutical agents used in this study are approved for use in food-producing animals and, from a public health perspective, do not pose a risk to consumers. We decided to use injectable anaesthetics, which are desirable in practice mainly because of their ease of use, as the use of inhalation anaesthetics is practically impossible in farmed animals. At the same time, we were aware that chosen injectable anaesthetics have several disadvantages, such as inhibition of the cardiovascular system, inability to adjust the dose after administration, and more frequent excitations during awakening, as also described by other authors [23,25]. The use of injection-delivered medications introduces an additional stress factor, which may influence animal welfare and, in extreme cases, potentially affect meat quality due to localised reactions at the injection site. No such adverse effects were observed; however, this remains an important consideration when working with food-producing animals.

Our results indicate that animal handling, particularly during the second CORT sampling—when handling was most intensive across all groups—led to a general elevation in stress hormone concentrations, with the except in group 8. This suggests that both handling injections and, in our case, blood sampling are inherently stressful procedures. Nevertheless, we consider that the benefits provided by anaesthesia, especially in reducing intraoperative pain and improving overall welfare, outweigh these temporary stress-related drawbacks. Future studies could aim to optimise administration techniques and minimise handling intensity to further reduce stress while preserving the welfare advantages of anaesthetic protocols. The statistical difference in CORT concentration was not confirmed between groups 7 and 8, where fasting was performed in group 7. We conclude that fasting in our study did not induce stress that could be detected.

Birds react to pain in a very specific way: initially, they attempt to escape, and if the painful stimulus continues, tonic immobility follows [57], which gives the impression that the animal is anaesthetised. This is probably also the reason that certain surgical procedures (e.g., beak shortening, flower removal, amputation of the hind tip of the inner fingers, coating, etc.) that would be considered painful in mammals are performed in birds without the use of anaesthesia and analgesia, as the animals show only mild or no signs of agitation at all [23,25]. This could lead to the misconception that procedures without anaesthesia are acceptable. However, findings from this study reveal several indicators demonstrating that caponisation—particularly when performed without anaesthesia—elicits behavioural responses and reactions consistent with pain. Therefore, surgical techniques must be adapted to the specific needs of each species, avoiding the assumption that what is technically feasible is ethically tolerable. Observations reveal that, despite the use of anaesthesia, some reactions still occurred in anaesthetised cockerels. However, high-grade reactions were noticeably reduced across all observed stages of the procedure.

As expected, there are confirmed differences between the anaesthetised and unanaesthetised groups. In the unanaesthetised groups, the data were uniform and indicated a 100% awake state of the animals, while in the anaesthesia groups, incomplete anaesthesia manifested as movements and more pronounced muscle tone.

In mammals, pain is processed in the cortex, but in birds, there is no clear equivalent of the cerebral cortex. Instead, the pain travels to the pallium, the part of the brain that corresponds to the neocortex in mammals [58]. Experimental studies have shown that birds display behavioural signs of pain, such as withdrawal, tremors, unnatural postures, and decreased activity. During pain, CORT is released, which is the main stress hormone in birds [19].

It is well known that alpha agonists do not work well under stress and are not effective [59]. This may be due to stress in preparation for the procedure, which prevented the cockerels from fully calming down and falling asleep.

By assessing the reaction to pain stimuli, we tried to evaluate the individual pain stimuli present during caponisation and to determine whether there was a difference between the group where the surgical procedure was performed without anaesthesia and analgesia (group 1) and those where caponisation was done with different combinations of anaesthesia and analgesia (groups 2–4).

Experimental and clinical evidence suggests that birds are capable of experiencing both somatic and visceral pain arising from internal organs such as the gastrointestinal tract and gonads, and that this pain is modulated through mechanisms analogous to those observed in mammals [60,61,62]. Based on these studies on visceral pain, we decided to monitor pain stimuli during rotation of the second testicle, which we determined as the peak pain point. Testicular rotation involves visceral pain. Additionally, the cumulative effect of preceding painful stimuli, such as feather plucking and incision, was expected to amplify pain perception at this stage. Visceral pain, in contrast to superficial stimuli, typically induces stronger autonomic and behavioural reactions.

Meloxicam is the most frequently used NSAID, apparently safe at a dose of 0.2 mg/kg to 3 mg/kg body weight (bw) [63]. In our study, we used a dose of 1.0 mg/kg bw and did not observe negative effects of meloxicam at the used dosage, but an analgesic effect manifested in the observed parameters was also not noticed.

We found that the unanaesthetised cockerels showed a greater response to the pain stimulus than the anaesthetised cockerels as shown in Table 6. Cockerels given only analgesics exhibited signs of pain at the same level as animals that did not receive any preparation. This is most likely due to the mechanism of action of the analgesic meloxicam, a nonsteroidal anti-inflammatory drug (NSAID). Its action is mainly aimed at inhibiting postoperative inflammation and the associated pain) [64], but it does not have an immediate effect on acute pain during the procedure. For more optimal results, it would probably be more effective to choose an analgesic with a direct effect on acute pain suppression (e.g., opioids). On the other hand, both components of anaesthesia, ketamine and xylazine, have analgesic properties of their own, which is also reflected in the lower percentage of reactions and vocalisations in the anaesthetised groups.

The combination of ketamine (a dissociative anaesthetic) and xylazine (an α2 adrenoceptor agonist) is widely used in veterinary medicine because their effects are complementary: ketamine provides rapid induction and analgesia, while xylazine contributes sedation, muscle relaxation, and additional analgesia. Together, they provide deeper and more stable anaesthesia than either drug alone [65,66].

At the time of use, both medications were labelled for administration in food producing animals. Although other combinations may be more effective [65], the anaesthetics investigated were chosen because they were readily available on the market, approved for other food-producing animals, practical, and economical for use in farm settings, where inhalational anaesthesia or other combinations may not be applicable.

We conclude that it is easier to perform the procedure with anaesthesia than in unanaesthetised cockerels. Anaesthesia therefore contributed positively to less painful and safer caponisation. At the same time, vocalisation, movement, and rapid breathing make it more difficult for the surgeon to perform the procedure, increasing the likelihood of errors that can cause discomfort or pain to the cockerel. We found that the procedure is easier to perform in anaesthetised cockerels (up to 10% difficulties) than in unanaesthetised cockerels (up to 40% difficulties). This is attributed to the absence or reduction in responses, which complicate the surgeon’s work. In the caponised group with analgesia only (group 3), the procedure was more difficult, which confirms the findings in other parameters.

Certain physiological parameters such as heart rate, breathing and body temperature are known to reach higher values under pain and stress, so they can be used for clinical evaluation and monitoring of pain in animals [67]. In our setting, we did not use body temperature as an indicator, as opening the body cavity, the use of antiseptics, and anaesthesia could ultimately lead to inconclusive results.

While these parameters are not specific to pain alone and also respond to other procedure-related stressors such as agitation, fear, restraint, and handling [57,67], they provide an integrated measure of the birds’ overall welfare. In this context, their responsiveness to multiple stressors can be considered a strength, as it allows assessment of the combined welfare impact of anaesthesia and analgesia during caponisation.

Although monitoring these parameters was feasible within the controlled conditions of this study, anaesthesia monitoring may present significant challenges in a clinical setting. While electrode placement is generally achievable, interpreting elevated heart rates on the ECG remains difficult. Additionally, monitoring respiratory rate is challenging, as handling and manipulation of the animal can interfere with readings.

In particular heart rate during the procedure did not show a response to stimuli and was only statistically affected by anaesthesia. One limitation here is the inherently high pulse rate, which could also be due to a stress response caused by handling.

The values of physiological parameters are also affected by anaesthesia, making a direct comparison of anaesthetised with unanaesthetised animals not entirely appropriate. Acknowledging this, we included a control group of uncaponised cockerels with anaesthesia and analgesia (group 5) in the study, which provided data only on the effect of anaesthesia on physiological parameters.

Breathing frequencies did not show statistically lower results in anaesthetised animals at rest; rather, breathing was influenced by caponisation, with frequency statistically higher in unanaesthetised groups.

As expected for heart rate, we found statistical differences between the anaesthetised and unanaesthetised groups, whereas the comparison between heart rate at rest and at the designated peak pain point did not differ statistically.

Caution should be exercised when interpreting the respiratory rate, considering that ketamine anaesthesia, as well as the combination of ketamine and xylazine, mildly inhibits the respiratory centre, but not to such an extent as to obscure the signs of pain [23]. In our study, breathing rates in anaesthetised and unanaesthetised animal did not differ between groups before the procedure at rest. Unanaesthetised cockerels had a significantly higher respiratory rate during the procedure than anaesthetised cockerels. This fact can also be an indicator that caponisation causes pain and stress.

Vocalisation can reveal much about the current psychophysical state of cockerels. Studies have shown a link between pain and increased vocalisations. It is believed that louder and more frequent sounds indicate pain, which should be distinguished from other types of vocalisation, such as those expressing hunger or seeking a mate [29,68].

Although vocalisations in poultry encompass a wide range of functionally distinct call types, some of which have been associated with specific affective states, the present study focused on vocalisation intensity and persistence as indicators of acute pain-related responses. Differentiation of specific vocalisation types was beyond the scope of the behavioural observation method used and was therefore not included. Future studies combining qualitative vocalisation analysis with intensity-based scoring may provide a more nuanced assessment of affective states associated with pain and distress [69].

In our study, vocalisation and physical reactions were most pronounced in unanaesthetised groups. These responses indicate nociceptive activation and distress. Anaesthetised groups showed less vocalisation and reduced motor reactions, confirming the analgesic efficacy of the applied protocols. The lower percentage of difficult procedures in these groups further supports the conclusion that anaesthesia improves handling conditions and reduces animal suffering. Behavioural indicators thus provide strong, observable evidence of procedural impact and welfare status.

There are multiple physiological responses to stress. It should not be assumed that only one parameter is sufficient to determine stress, whether it is plasma concentrations of CORT, the heterophil/lymphocyte ratio, or a behavioural response [51]. Physiological and behavioural indices of stress are very useful as they can be quantified and generally align with established views of welfare.

In broilers, during periods of heat stress, plasma CORT values ranged from approximately 4 ng/mL to 30 ng/mL. Broilers from different systems (discharge group and discharge-free group) were compared. Their results support the claim that environmental conditions and the rearing system affect CORT concentration [70]. In our study, the concentration of CORT ranged from 0.1 to 6.8 ng/mL. This concentration pertains to samples from group 8, where only blood was collected without fasting, fixation, administration of drugs, or surgery. The maximum CORT concentration, 10.6 ng/mL, was found in group 4 (anaesthetics and analgesics were administered). The distribution of CORT concentrations across all groups and sampling points indicates that these results alone cannot reliably determine the stress response, as handling and sampling procedures are likely to act as confounding stressors that may mask the primary stress response induced by caponisation.

In our study, CORT levels peaked during the second sampling in all groups except groups 7 and 8, reflecting a response to procedural stress. Although anaesthetised animals exhibited fewer behavioural signs of distress, their CORT levels still rose significantly, indicating that anaesthesia does not fully suppress the internal endocrine stress response. The decline in levels during the third sampling suggests physiological recovery or adaptation.

These findings highlight the importance of interpreting hormonal data alongside behavioural and physiological parameters, as stress responses are influenced not only by pain but also by other external factors. One possible explanation for the elevated CORT levels across groups during the second sampling is that all cockerels were placed in boxes prior to the procedure or blood sampling. While this was also the case during the first and third sampling intervals, the time spent in boxes was likely longer during the second sampling, as the caponisation procedure took more time than blood sampling alone. Additionally, the caponisation process involved more movement within the housing and required more personnel, which may have contributed to increased stress.

In conclusion, although CORT is a useful marker of stress, it is only partially reliable for estimating pain. Other factors appear to play an equally significant role in the stress response. Point of pain designation: Multiple lines of evidence confirm removal of the second testis by rotation as the peak pain point during caponisation. Respiratory rate was highest at T2 across all treated groups (median = 48), with statistically significant differences between anaesthetised and non-anaesthetised cockerels (*p* < 0.05). Behavioural reactions at T2 were pronounced, with a high percentage of cockerels in unanaesthetised groups showing moderate to strong responses (grades 2 and 3). Additionally, the percentage of difficult procedures was highest in cockerels lacking anaesthesia, indicating increased resistance and distress. In contrast, heart rate showed high variability and did not consistently peak at the designated peak pain point, suggesting lower sensitivity under these conditions. The convergence of respiratory, behavioural, and procedural indicators at this point validates its selection as the focal point for pain assessment.

Evidence regarding the nociceptive effect of analgesic drugs in chickens is still fragmentary, and more research is needed to produce an objective assessment of pain and pain relief in chickens. In the absence of clear guidelines and due to limited available information, it is essential to follow good clinical practice and incorporate up-to-date data into future research and clinical procedures.

The continuation of caponisation—particularly surgical methods without pain management—may face tighter restrictions or even phase-outs in the coming years. Conversely, if carried out humanely and transparently, it could find a place in niche, premium markets where consumers are willing to pay more for ethical and high-quality meat.

## 5. Conclusions

Based on the elevated physiological parameters, vocalisations, and reactions, the results of the study confirm the hypothesis that caponisation is painful for cockerels. We concluded that anaesthesia contributes to pain reduction and facilitates the procedure, which is crucial for animal welfare. The use of NSAIDs, in our case meloxicam, did not provide significant pain reduction during caponisation. Furthermore, we did not confirm that the procedure had significant effect on weight gain six days after caponisation across groups.

## Figures and Tables

**Table 1:** Caponisation procedure–experimental groups (N = 72).

	Group 1	Group 2	Group 3	Group 4	Group 5	Group 6	Group 7	Group 8
Number of cockerels	10	10	10	10	8	8	8	8
Fasting	yes	yes	yes	yes	yes	yes	yes	no
Fixation on the table	yes	yes	yes	yes	yes	yes	no	no
Caponisation	yes	yes	yes	yes	no	no	no	no
Anaesthesia ^1^	no	yes	no	yes	yes	no	no	no
Analgesia ^2^	no	no	yes	yes	yes	no	no	no

^1^ Xylazine (Chanelle Pharma Group, Galway, Ireland) (i/m 3 mg/kg–0.15 ml/kg) and Ketamine Vetquinol (Towcester UK Ltd., Towcester, UK) (i/m 25 mg/kg–0.25 mL/kg); Xylazine and ketamine were administered together, mixed in a single syringe. All chickens in groups 2, 4 and 5 received the same combination of anaesthetics in a single application. ^2^ Meloxicam–Meloxidyl^®^ (Ceva Sante Animale Romania, Bucharest, Romania) (i/m 1 mg/kg–0.2 mL/kg).

**Table 2:** Operational scoring system based on movement intensity.

Grade	Reaction	Operational Definition
0	No response	No observable change in movement.
1	Mild response	Small movements of the wings or legs; the animal’s overall body position remains stationary.
2	Moderate response	Clear movements of the wings or legs that result in a small displacement or shift in the animal’s body position, no additional fixation is needed.
3	Strong resistance	Intense kicking, vigorous wing flapping, or escape attempts that require additional manual fixation to maintain the procedure.

**Table 3:** Weight gain and feed intake in the different groups (N = 72).

	Group 1	Group 2	Group 3	Group 4	Group 5	Group 6	Group 7	Group 8
Median mass at day of caponisation (g)	692 *	706 *	670 *	719 *	697 *	710 *	721 *	779 *
IQR (25–75%)	666–720	664–736	655–692	694–731	669–735	687–728	687–746	766–840
Significant difference between groups (*p* ≤ 0.05)	8 **	8 **	8 **	8 **	8 **	8 **	8 **	All **
Median mass at day 7 after caponisation (g)	927 *	927 *	906 *	947	966 *	968 *	967 *	997 *
IQR (25–75%)	896–943	875–972	877–934	911–959	886–1003	945–990	944–996	955–1050
Significant difference between groups (*p* ≤ 0.05)	ns **	ns **	ns **	ns **	ns **	ns **	ns **	ns **
Median feed intake day 1–6 after caponisation) (g)	86 *	86 *	107 *	79 *	111 *	114 *	106 *	102 *
IQR (25–75%)	82–96	78–98	98–116	76–84	108–113	108–117	100–108	95–1056
Significant difference between groups (*p* ≤ 0.05)	6 ***	5, 6 ***	ns ***	5, 6 ***	4, 2 ***	4, 2, 1 ***	ns ***	ns ***

* Data in the group follow a normal distribution at sampling; values are presented as medians for consistency across groups. ** Parametric test applied; overall normality across all compared data was confirmed. *** Non-parametric test applied; overall normality across compared data not confirmed. ns No statistically significant difference (*p* > 0.05).

**Table 4:** The respiratory rate of cockerels in the different groups at the control points, in inspirations per minute.

	Group 1	Group 2	Group 3	Group 4	Group 5	Group 6
Average breathing at rest (min^−1^)	31 *	32 *	39 *	34 *	30 *	39 *
Standard Deviation (SD±)	7	5	5	9	8	7
Significant difference between groups (*p* ≤ 0.05)	ns **	ns **	ns **	ns **	ns **	ns **
Average breathing at designated peak pain point (min^−1^)	62 *	30 *	63 *	31 *	na	na
Standard Deviation (SD±)	9	7	13	8	na	na
Significant difference between groups (*p* ≤ 0.05)	2, 4 **	3, 1 **	2, 4 **	3, 1 **	na	na
Difference in breathing frequency between observed points (*p* ≤ 0.05)	yes **	no **	yes ***	no **	na	na

* Data in the group follow a normal distribution at sampling. ** Parametric test applied, as overall normality across all compared groups was confirmed. *** Non-parametric test applied; overall normality across compared data not confirmed. ns—No statistically significant difference (*p* > 0.05). na—Not applicable.

**Table 5:** Heart rate of cockerels in the different groups at the control points.

	Group 1	Group 2	Group 3	Group 4	Group 5	Group 6
Median heart rate at rest (min^−1^)	420 *	300	420 *	300 *	347 *	420
IQR (25–75%)	411–480	240–300	420–446	300–360	300–373	420–420
Significant difference between groups (*p* ≤ 0.05)	2, 4, 5 **	1, 3, 6 **	2, 4, 5 **	1, 3, 6 **	1, 3 **	2, 4 **
Median heart rate at designated peak pain point (min^−1^)	420	317 *	425 *	360 *	na	na
IQR (25–75%)	360–420	300–360	420–463	300–360	na	na
Significant difference between groups (*p* ≤ 0.05)	2, 4 **	1, 3 **	2, 4 **	1, 3 **	na	na
Difference in heart rate between observed points (*p* ≤ 0.05)	no **	no ***	no **	no **	na	na

* Data in the group follow a normal distribution at sampling; values are presented as medians for consistency across groups. ** Parametric test applied, as overall normality across all compared groups was confirmed. *** Non-parametric test applied; overall normality across compared data not confirmed. na—not applicable.

**Table 6:** Assessment of vocalisation during caponisation.

Percentage of Difficult Procedures (%)															
Group 1				Group 2				Group 3				Group 4			
30.0				0.0				40.0				10.0			
Reaction at designated peak pain point (%) *															
Group 1 (reaction grade *)				Group 2 (reaction grade *)				Group 3 (reaction grade *)				Group 4 (reaction grade *)			
0	1	2	3	0	1	2	3	0	1	2	3	0	1	2	3
0.0	20.0	50.0	30.0	11.0	11.0	56.0	22.0	0.0	20.0	30.0	50.0	0.0	33.0	44.0	22.0
Reactions across all measured points during caponisation (%) *															
Group 1 (reaction grade *)				Group 2 (reaction grade *)				Group 3 (reaction grade *)				Group 4 (reaction grade *)			
0	1	2	3	0	1	2	3	0	1	2	3	0	1	2	3
8.3	31.7	33.3	26.7	55.9	27.1	10.2	6.8	13.3	33.3	26.7	26.7	52.2	25.4	16.9	5.1
Vocalisation at designated peak pain point (%) **															
Group 1 (reaction grade **)				Group 2 (reaction grade **)				Group 3 (reaction grade **)				Group 4 (reaction grade **)			
0	1	2	3	0	1	2	3	0	1	2	3	0	1	2	3
70.0	10.0	10.0	10.0	90.0	0.0	0.0	10.0	70.0	0.0	30.0	0.0	100.0	0.0	0.0	0.0
Vocalisations across all measured points during caponisation (%) **															
Group 1 (reaction grade **)				Group 2 (reaction grade **)				Group 3 (reaction grade **)				Group 4 (reaction grade **)			
0	1	2	3	0	1	2	3	0	1	2	3	0	1	2	3
61.7	6.7	8.3	23.3	91.7	1.7	0.0	6.7	65.0	10.0	10.0	15.0	90.0	3.3	5.0	1.7

* 0—no response; 1—mild kicking or wing flapping; 2—moderate kicking or wing flapping; 3—strong resistance and escape attempts; ** 0—no vocalisation; 1—quiet, short vocalisation; 2—loud, longer vocalisation; 3—very loud vocalisation, lasting long after the stimulus.

**Table 7:** CORT concentration (ng/mL blood serum) in the different groups (N = 63).

	Group 1	Group 2	Group 3	Group 4	Group 5	Group 6	Group 7	Group 8
CORT concentration— Sampling 1	0.868 *	0.726 *	1.699 *	2.090 *	1.570 *	0.528 *	0.879 *	1.075 *
IQR (25–75%)	0.459–1.444	0.342–2.929	0.989–1.994	0.719–2.412	1.114–1.672	0.379–0.982	0.335–1.548	0.828–1.311
Significant difference between groups (*p* ≤ 0.05)	ns **	ns **	ns **	ns **	ns **	ns **	ns **	ns **
CORT concentration— Sampling 2	4.496 *	5.649 *	5.810 *	6.076 *	3.916 *	3.766 *	3.220 *	3.164 *
IQR (25–75%)	3.758–6.723	2.312–6.845	4.359–7.253	2.440–8.602	2.775–4.348	2.096–4.175	2.944–3.908	1.742–4.701
Significant difference between groups (*p* ≤ 0.05)	ns **	ns **	ns **	ns **	ns **	ns **	ns **	ns **
CORT concentration— Sampling 3	0.765	1.478 *	1.178 *	1.620	3.245 *	1.811 *	1.956 *	1.960 *
IQR (25–75%)	0.319–1.422	0.674–2.050	0.546–2.134	1.044–3.122	2.707–4.151	0.814–3.579	0.869–3.422	1.264–3.918
Significant difference between groups (*p* ≤ 0.05)	5 > 1 ***	ns ***	ns ***	ns ***	5 > 1 ***	ns ***	ns ***	ns ***
Significant difference between samplings (*p* ≤ 0.05)	2 > 1, 3 **	2 > 1, 3 ***	2 > 1, 3 **	2 > 1, 3 **	2, 3 > 1 **	2 > 1 ***	2 > 1 ***	ns **

* Data in the group follow a normal distribution at sampling; values are presented as medians for consistency across groups. ** Parametric test applied, as overall normality across all compared groups was confirmed. *** Non-parametric test applied; overall normality of compared data not confirmed. ns No statistically significant difference (*p* > 0.05).

## Data Availability

Data can be provided upon request.

## Abbreviations

The following abbreviations are used in this manuscript:

ANOVA	Analysis of Variance
CORT	Corticosterone
EC	Commission Regulation
ECG	Electrocardiogram
EU	European Union
(F)	Feather plucking
GnRH-immunocastration	Gonadotropin-Releasing Hormone Immunocastration
HPA	hypothalamic–pituitary–adrenal
(I)	Skin incision
IQR	Interquartile range
NSAIDs	Non-steroidal anti-inflammatory drugs
(O)	Abdominal wall opening
(R)	Rest
(T)	Testicular rotation
